# The association between dietary quality scores with C-reactive protein and novel biomarkers of inflammation platelet-activating factor and lipoprotein-associated phospholipase A2: a cross-sectional study

**DOI:** 10.1186/s12986-023-00756-x

**Published:** 2023-09-12

**Authors:** Carolyn J. English, Anna E. Lohning, Hannah L. Mayr, Mark Jones, Helen MacLaughlin, Dianne P. Reidlinger

**Affiliations:** 1 Faculty of Health Sciences and Medicine, Bond University, Robina, QLD Australia; 2 Department of Nutrition and Dietetics, Princess Alexandra Hospital, Woolloongabba, QLD Australia; 3 Centre for Functioning and Health Research, Metro South Hospital and Health Service, Brisbane, QLD Australia; 4 Faculty of Health Sciences and Medicine, Institute of Evidence-Based Healthcare, Bond University, Robina, QLD Australia; 5 Faculty of Health, School of Exercise and Nutrition Sciences, Queensland University of Technology, Brisbane, Australia; 6 Nutrition Research Collaborative, Royal Brisbane and Women’s Hospital, Brisbane, Australia

**Keywords:** Inflammation, Cardiovascular diseases, Diet healthy, Diet vegetarian, Dietary approach to stop hypertension, Biomarker, Diet Quality, COVID-19

## Abstract

Healthy dietary patterns are associated with lower inflammation and cardiovascular disease (CVD) risk and adherence can be measured using diet quality scores. Inflammation is traditionally measured with C-reactive protein (hsCRP), however there is interest in novel pro-inflammatory markers platelet-activating factor (PAF) and lipoprotein-associated phospholipase A2 (Lp-PLA_2_) that are specifically involved in endothelial dysfunction and inflammation. This cross-sectional study investigated the association between PAF, Lp-PLA_2_, hsCRP, and six diet scores. One hundred adults (49 ± 13 years, 31% male) with variable CVD risk were recruited. Fasting PAF, Lp-PLA_2_ and hsCRP and usual dietary intake were measured. Adherence to Dietary Approaches to Stop Hypertension (DASH), Dairy-adjusted DASH, Vegetarian Lifestyle Index, Healthy Eating Index for Australians (HEIFA), Mediterranean Diet Adherence Screener (MEDAS) and PREDIMED-Plus (erMedDiet) scores were calculated. Correlations and multiple regressions were performed. hsCRP, but not PAF, independently correlated with several diet scores. Lp-PLA_2_ independently correlated with Vegetarian Lifestyle Index only in unadjusted models. A one-point increase in adherence to the DASH Index, the Dairy-adjusted DASH Index and the Vegetarian Lifestyle Index was associated with a 30%, 30%, and 33% reduction in hsCRP levels, respectively. Smaller effects were seen with the other diet scores with a one-point increase in adherence resulting in a 19%, 22% and 16% reduction in hsCRP with HEIFA, MEDAS, erMedDiet scores, respectively. The lack of stronger associations between the novel markers of inflammation and diet scores may be due to confounding by COVID-19 infection and vaccination programs, which prevents any firm conclusion on the relationship between PAF, Lp-PLA_2_ and healthy dietary patterns. Future research should aim to examine the relationship with these novel markers and healthy dietary patterns in a non-pandemic setting.

**Supplementary Information:**

The online version contains supplementary material available at 10.1186/s12986-023-00756-x.

## Introduction

Vascular inflammation plays a key role in atherosclerosis formation and progression resulting in the development of cardiovascular disease (CVD) [1]. Individual dietary components have been shown to modulate inflammation, [2, 3] however, research on dietary patterns better captures how people eat, and the synergy that exists between individual foods when combined in an overall diet [4]. Diet quality scores have been developed to measure the healthfulness of diets and can be used to assess adherence to specific heathy dietary patterns. Higher quality diets, measured by several diet quality indices, are associated with a lower risk of CVD and all-cause mortality [5].

Numerous inflammatory biomarkers have been identified to detect and measure inflammatory processes involved in atherosclerosis, one of which is high-sensitivity C-reactive protein (hsCRP). However, this is a non-specific marker of inflammation and can be elevated with both acute and chronic inflammation [6]. CRP has numerous isoforms, some more atherogenic than others, which current assays are not able to differentiate well [7]. In addition CRP has significant intraindividual variability and requires repeated measurements for accurate assessment [8].

Two novel biomarkers related to endothelial damage that have been identified in atherosclerosis research are platelet-activating factor (PAF) and lipoprotein-associated phospholipase A2 (Lp-PLA_2_) [9]. PAF is an ether-linked glycerophospholipid and is a potent inflammatory mediator involved in endothelial dysfunction and permeability, cell signalling and initiation of the inflammatory cascade within the intima [10]. PAF has been shown to be associated with numerous CVD events including myocardial infarction, heart failure, stroke and coronary heart disease [11–14].

Lp-PLA_2_ is a vascular specific marker with a low biologic fluctuation that is involved in plaque growth and thrombosis [15]. Lp-PLA_2_ hydrolyses the *sn*-2 ester bond of glycerophospholipids such as the acetyl group on PAF, in addition to oxidised phospholipids on the surface of LDL particles [16]. Elevated levels of Lp-PLA_2_ have been shown to be indicative of vascular inflammation associated with the formation of plaque within the arteries [17] and is involved in several CVD events including stroke, myocardial infarction, coronary artery disease and aortic stenosis [18–21].

Compliance with healthy dietary patterns such as Dietary Approach to Stop Hypertension (DASH) diet, Mediterranean diet, vegetarian diet and diets based on country specific dietary guidelines are associated with lower levels of inflammatory biomarkers and CVD risk [22–25]. However, research examining dietary patterns and inflammation measured by PAF and Lp-PLA_2_ is limited. A recent systematic review of 16 studies found Mediterranean diet scores, vegetarian diets and heart healthy diets to be associated with lower levels of PAF and Lp-PLA_2_ [26]. However, most of the included studies had numerous unadjusted confounders such as the inclusion of participants on medications and/or supplements known to lower levels of these markers such as cholesterol-lowering medications, hormone replacement therapy, niacin, orlistat, fish oils and omega-3 supplements [27–32].

Therefore, the aim of this study was to examine the relationship between hsCRP, PAF and Lp-PLA_2_ activity and healthy dietary patterns utilising strict exclusion criteria. Specifically, this study aimed to investigate the relationship between these novel markers of inflammation and hsCRP, and six diet quality scores including DASH Index, Dairy-adjusted DASH Index, Vegetarian Lifestyle Index, Healthy Eating Index for Australian Adults (HEIFA), original Mediterranean Dietary Adherence Screener (MEDAS), and modified MEDAS from the PREDIMED-Plus Study (erMedDiet) in Australian adults at varied levels of CVD risk.

## Material and methods

Methodology for this study, excluding the assessment of dietary intake and calculation of dietary scores, has been previously published [9].

### Study design and setting

This study used a cross-sectional design and a convenience sampling technique and was carried out on the Gold Coast, Queensland, Australia. Participants were recruited through non-health community settings such as sporting clubs, surf life savings clubs, shopping centres, fitness centres, council libraries, community centres, university setting and through social media and online/email methods to obtain a representative community sample of healthy adults at varying risk of cardiovascular disease. Recruitment for the study began in February 2021 and samples were collected from May 2021 to April 2022, over four two-week periods.

This study protocol was approved by the Bond University Human Research Ethics Committee (approval DR03194) and conforms to the ethical guidelines of the 1975 (revised in 1983) Declaration of Helsinki. All participants provided written informed consent.

### Study population and sample size

Eligible participants included adults aged 18–70 years old who were classified as either low or high risk of CVD. In order to obtain a more robust estimate of the relationship between diet quality and inflammatory markers we aimed to get variety in CVD risk from low to high. In order to be classified as high risk of CVD, participants had to either have confirmed type 2 diabetes OR have two or more of the following risk factors for CVD: systolic blood pressure ≥ 140 mm Hg or diastolic ≥ 90 mm Hg or receiving medication for high blood pressure; total cholesterol ≥ 5.2 mmol/L; LDL cholesterol ≥ 4.1 mmol/L; HDL cholesterol ≤1 mmol/L; family history of premature CHD (≤ 60 years); or excess weight, BMI ≥ 25 kg/m^2^. To be classified low risk, participants had to report the absence of any chronic disease and not be on any routine medication. They also needed to be below the cut-offs listed for high-risk individuals for BMI, blood pressure, cholesterol and report no family history of premature CHD.

Exclusion criteria included any participant who reported a history of angina, myocardial infarction, peripheral vascular disease, congenital heart disease or stroke or who were current smokers. Additionally, any participant who was taking medications or supplements known to impact measurements of PAF and/or Lp-PLA_2,_ including cholesterol lowering medications such as statins, fenofibrate, and ezetimibe; or niacin, orlistat, omega- 3, fish oil supplements and hormone replacement therapy were excluded. Participants who reported Asian or African ethnicity were also excluded as these ethnic groups have been shown in studies to have lower levels of Lp-PLA_2_, possibly due to genetic polymorphisms [33–35].

Similar studies with PAF and/or Lp-PLA_2_ as outcome measures have used sample sizes ranging from 10 to 106 participants [36–39]. With 100 participants there was an 80% power to detect a correlation between inflammation level and diet scores of 0.3 or greater assuming a level of significance of 5%. A correlation of 0.3 is a medium effect size for a correlation according to Cohen [40].

### Data collection

Data were collected at the Bond Institute of Health and Sport through face-to-face visits and included anthropometric, biochemical, and clinical measurements.

Anthropometric data were measured with normal clothing but without shoes and in the fasted state. A wall mounted stadiometer was used to measure standing height to the nearest 0.1 cm. Weight was measured to the nearest 0.1 kg with a calibrated digital scale. Waist circumference was measured six times, three times at minimum waist and three times at the umbilicus and was averaged [41]. The formula (weight (kg)/height (m^2^) was used to calculate BMI.

Clinic blood pressure was measured in triplicate, two minutes apart, in the non-dominate arm, and seated with a clinical cuff using a Creative Medical PC-900 Pro Vital Signs Monitor. The first measurement was disregarded and the second and third measurements were averaged [42].

Participants completed self-administered questionnaires at the study visit which gathered information on age, sex, medical history, medication and supplement intake, menopausal status, smoking status and alcohol consumption.

The World Health Organization’s (WHO’s) Global Physical Activity Questionnaire (GPAQ) was used to measure physical activity (PA) levels of the participants [43]. The questionnaire was self-administered and included 16 questions assessing time spent physically active during work, travel, and recreation in addition to sedentary time. Participants’ metabolic equivalent (MET) minutes per weeks were then calculated based on the participant scores in accordance with the GPAQ Analysis Guide [44]. PA levels were categorised into tertiles using the MET minutes based on WHO’s PA recommendation where 0 = low, MET < 600 min/week; 1 = moderate, MET ≥ 600 to < 1500 min/week; and 2 = high, MET ≥ 1500 min/week.

Methods for blood collection and treatment and assay procedures for hsCRP, PAF and Lp-PLA_2_ were previously described and reported elsewhere [9].

### Dietary assessment

The usual dietary intake of participants was assessed by administering the European Prospective Investigation into Cancer and Nutrition (EPIC) food frequency questionnaire (FFQ) [45] modified for the Australian food environment. The EPIC FFQ is a validated tool developed to measure habitual food and nutrient intake in adults and children during the past year. This FFQ is a semi-quantitative paper-based questionnaire that includes 130 common and less common food items in addition to an open section where participants can add additional items not assessed in the questionnaire such as breakfast cereal brand, type and quantity of milk consumed, type of fat used in cooking and baking and the amount of visible fat on meat consumed. Participants responded by reporting the consumption frequency of each food item using a 9-point scale from never or less than once a month to 6 or more times per day. The frequency of each food item was manually entered into a spreadsheet, multiplied by the portion fraction based on frequency and multiplied by the quantified serving size of the food in grams, and was further converted into serving sizes.

In order to calculate energy intake and nutrient intake such as saturated and unsaturated fats and sodium that were needed to calculate some of the diet scores, the FFQ EPIC Tool for Analysis (FETA) software was utilised. The FETA software is an open source, cross-platform tool that processes dietary data from the food frequency questionnaire used by the EPIC-Norfolk [46]. The software contains 10 data files that contain all the individual foods, nutrients, and serving sizes based on European food composition data. Adaptations were made to the original FETA files to replace the European food composition data with the Australian Food Composition Database and AUSNUT values [47, 48]. This involved manually replacing all food items and each food item’s nutrients (energy, fat, carbohydrate, protein, and potassium, phosphorus, and sodium) according to the Australian Food Composition Database.

Participants completed an estimated three-day food diary in household measures the week following their study visit. The diaries were completed on 2 consecutive weekdays and a weekend day. This diary was used to calculate water intake for some of the diet scores.

### DASH Index

The adherence to the DASH dietary pattern was assessed by a DASH diet score designed by Gunther et al [49]. This score is comprised of 10 components to assess the participant’s compliance to the DASH dietary pattern (see Additional file 1: Table S1). Six of the components were weighted on a 10-point scale, which include: fruits and fruit juice; vegetables and potato; meat, poultry, fish and eggs; nuts, seeds and legumes; fats and oils; sweets.

Two additional components are grain and dairy and are assessed based on a qualitative aspect in addition to a quantitative one. Each is weighted on a 5-point scale and include: total dairy; low-fat dairy; whole grains; high-fibre grains.

Each participant was assigned the energy level that was closest to their estimated energy requirement based on age, sex, and physical activity level [50] and categorised into 1600, 2000, 2600 or 3100 cal as outlined in the National Heart, Lung and Blood Institute’s Dash Eating Plan [51]. Each dietary component was then standardised to the assigned energy level. Lower intakes were scored proportionally. Reverse scoring was applied where lower intakes are favoured by DASH, such as meats, poultry fish, eggs, fats, oils and sweets. The total DASH score was calculated by summing all the points from each respective section to generate a composite score ranging in values from 0 to 80.

### Dairy-Adjusted DASH Index

As recent research has highlighted the anti-inflammatory capabilities of full fat dairy [52] and that a DASH diet with full fat dairy consumption may be just as effective as low fat dairy in reducing blood pressure and lipids in clinical trials, [53] a sub analysis was performed to assess the relationship of the DASH index (with an adjustment for the dairy component) on inflammatory markers. This adjustment was performed by removing the scoring component for 75% of dairy consumed to be low fat.

### Vegetarian Lifestyle Index

Adherence to a vegetarian dietary pattern was assessed using the Vegetarian Lifestyle Index, a score developed to measure adherence to the Loma Linda University Vegetarian Food Guide Pyramid [54]. This score measures adherence to both diet and lifestyle recommendations. The score is comprised of fourteen components with eleven measuring aspects of diet and three measuring compliance to lifestyle habits (see Additional file 1: Table S2). The dietary components assessed included: whole grains; legumes and soy; vegetables; fruits; nuts and seeds; vegetable oils; dairy products; eggs; sweets; reliable sources of vitamin B-12; flesh foods. The following lifestyle habits were also assessed: daily exercise; water intake; adequate sunlight exposure.

Each dietary component was standardised to 2000 kcal (8368 kJ) per day specific to the lacto-ovo recommendations of the Vegetarian Food Guide Pyramid Guidelines for Healthful Vegetarian Diets [54]. This adjustment was based on BMR multiplied by a physical activity factor to standardised energy intake [50]. Each component was scored with 0, 0.5, or 1 point depending on adherence, with lower intakes receiving lower scores. Reverse scoring was applied where lower intakes are favoured by the vegetarian diet, such as dairy, eggs, sweets, and flesh foods. Daily exercise was assessed using the World Health Organization’s Global Physical Activity Questionnaire. All participants received full marks for sunlight exposure due to the location of this study as it has been shown that, even in dark skinned people, adequate vitamin D is produced in as little as 4 min of daily exposure to sunlight in winter, for people living in southeast Queensland [55]. The total vegetarian score was calculated by summing all of the points from each respective section to generate a composite score ranging in values from 0 to 14.

### HEIFA Index

Adherence to the Australian Guide to Healthy Eating (AGHE) was assessed using the HEIFA index [56]. This score is comprised of 11 components based on different characteristics of a healthy diet (see Additional file 1: Table S3). The degree to which each participant’s diet conformed to the serving recommendations for each food group of the AGHE was measured by each component. These components included the five core food groups, which include: vegetables; fruits; grain (cereal) foods; milk and milk alternatives; meat and protein food alternatives.

Three of the food groups, vegetables, fruits and grains, were assessed based on a qualitative aspect in addition to a quantitative one. Fruits and vegetables were assessed for variety and grains were assessed on whether 50% of total grains consumed are wholegrains.

One component assessed intake of discretionary foods which were high in saturated fat and/or added sugars, added salt, or alcohol. Additional components included alcohol intake and adequate water consumption. Water was assessed as a percentage of water to total beverages where 50% water consumed relative to total beverages received a full score.

Specific nutrients were assessed as separate components and include fats such monounsaturated, polyunsaturated and saturated fats, added sugars, and sodium. Saturated fat intake was assessed as a percentage of energy intake, with an intake of less than 10% required for a full score and a 0 was given when the ratio was 15% or greater. Nine of the components (core food groups, discretionary foods, saturated fat, added sugar and sodium) were scored from 0–10. Two components (water and alcohol) were scored from 0 to 5. All components were summed to reach a composite score which ranged from 0 to 100.

### Mediterranean diet adherence screener (MEDAS)

A 14-item MEDAS tool was used to assess adherence to the Mediterranean Diet [57]. Five questions assessed intake of favourable food groups such as fruit, vegetable, legumes, fish and shellfish, nuts (see Additional file 1: Table S4). Two questions assessed olive oil intake, both quantity and whether olive oil was used as the main cooking oil. One question assessed how many times per week boiled vegetables, pasta, rice, or other dishes were consumed with a sofrito sauce of tomato, garlic, onion, or leeks sauteed in olive oil. Four questions assessed intake of foods that are not favourable in the Mediterranean Diet such as red and processed meat, sugar sweetened beverages, butter, margarine or cream intake, and consumption of commercial sweets and pastries. One question assessed whether chicken and turkey meat were preferentially consumed over red meat, pork and processed meat. One question assessed wine intake. Each question had a single adherence criterion and was worth 1 point if that criteria was met, or 0 points if not met. Scores were summed with a total composite score ranging from 0 to 14.

### erMedDiet Score

Adherence to the Mediterranean Diet was also assessed using the 17-item erMedDiet score [58]. This score is an adapted version of the original 14-item MEDAS that is based on a randomised control trial of the Mediterranean diet that allowed for energy restriction and body mass reduction. Similar to the original MEDAS, five questions assessed intake of favourable food groups such as fruit, vegetable, legumes, fish and shellfish, nuts (see Additional file 1: Table S5). One question assessed whether olive oil was used as the main cooking oil. One question assessed how many times per week boiled vegetables, pasta, rice, or other dishes were consumed with a sofrito sauce of tomato, garlic, onion, or leeks sauteed in olive oil. Three questions assessed grain intake with one question assessing whole grain intake and two questions assessing refined grains and white bread. Four questions assessed intake of foods that are not favourable in the Mediterranean Diet such as red and processed meat, sugar sweetened beverages, butter, margarine or cream intake, and consumption of commercial sweets and pastries. One question assessed whether chicken and turkey meat were preferentially consumed over red meat, pork and processed meat. One question assessed whether sugar was added to coffee and tea and one question assessed wine intake.

Each question was scored using the same method as the MEDAS and scores were summed with a total composite score ranging from 0 to 17. For questions based on the same components as the MEDAS some were adjusted with regards to their frequency/serve intake criteria to accommodate energy restriction.

### Data analysis

All data were analysed using SPSS version 28.0.0.0 (190) (SPSS Inc., Chicago, USA). Data were assessed for normality by examining distributions via Q–Q plots. Variables that were not normally distributed were log transformed before data analysis (PAF and hsCRP). Independent t-tests were performed on normally distributed variables to test for differences between males and females, and participants with high-risk and low-risk of CVD. Pairwise Pearson’s correlations were calculated between each of the six measured diet scores as well as between the diet scores and markers of inflammation.

Multiple linear regression was performed to examine associations between markers of inflammation and diet scores. A first model was run that adjusted for age, gender and year of data collection. A second model adjusted for age, gender, year of data collection, waist circumference, physical activity and level of risk. Models which examined the Vegetarian Lifestyle Index were not adjusted for physical activity as this was accounted for in the formulation of the Vegetarian Lifestyle Index score. Checks for multicollinearity were conducted using variance inflation factor (VIF) and tolerance indices and revealed no evidence of multicollinearity. Results of multiple linear regression are reported as standardised coefficients ß and significance is reported as *P* values where *P* < 0.05 is considered statistically significant. Mean scores for each inflammatory marker concentration were calculated for quartiles of each of the diet scores for descriptive purposes only and were not used in regression analysis (see Additional File 1: Table S7).

To estimate the effect of a one-point change in diet scores that were reported as statistically significant in model 1, the β coefficients were back transformed by exponentiating the coefficient. This allowed interpretation on a multiplicative scale, e.g., a back transformed value of 0.70 means a 1 unit increase in diet score is associated with a 1–0.70 = 30% decrease in the inflammation measure.

To explore the effects that year of data collection (and potential COVID-19 illness and/or vaccine) had on results for PAF and Lp-PLA_2_, a separate multiple regression was performed (Table 5).

## Results

### Clinical characteristics

A total of 132 people were recruited; four did not meet inclusion criteria and 28 declined to participate, leaving 100 participants who attended a study data collection visit and were included in analysis (Additional file 1: Table S6). Forty-six participants (44 high-risk, 2 low-risk) attended study visits in 2021 and 54 participants (24 high-risk and 30 low-risk) attended in 2022. Demographic and clinical characteristics for the total cohort, males and females, and individuals at high- versus low- CVD risk are shown in Table 1. The mean age was 49 (range 20–69) years and 92% of the cohort were Caucasian. Mean HDL cholesterol was 1.84 ± 0.48 mmol/L and was significantly higher in females compared to males. Mean LDL cholesterol was 3.16 ± 1.11 mmol/L and triglycerides were 1.40 ± 0.82 mmol/L.

**Table 1:** Demographic and Clinical Characteristics of Study Subjects

	Mean ± SD or N (%) or Median (IQR range)				Mean ± SD or N (%) or Median (IQR range)		
Characteristics	Total n = 100	Male n = 31	Female n = 69	*P value*^*a*^	High risk of CVD n = 68	Low risk of CVD n = 32	*P value*^*a*^
Age, years^b^	49 ± 13	46 ± 13	50 ± 13	0.120	53 ± 13	38 ± 14	** < 0.001**
Race, Caucasian n (%)	92 (92)	25 (86)	67 (94)	−	65 (96)	27 (84)	–
Male n (%)				−	21 (31)	10 (31)	–
BMI, kg/m^2b^	28.3 ± 6.5	27.41 ± 5.0	28.65 ± 7.2	0.729	30.65 ± 6.4	23.19 ± 2.7	** < 0.001**
Waist Circumference (cm) Umbilicus^b^	95.8 ± 6.7	95.99 ± 12.60	95.70 ± 18.40	0.526	102.36 ± 15.40	81.83 ± 9.15	** < 0.001**
Type 2 Diabetes diagnosis %	4 (4)	3 (10)	1 (1)	–	4 (6)	0 (0)	–
Physical Activity METs tertiles	1.41 ± 0.65	1.61 ± 0.72	1.32 ± 0.83	0.193	1.28 ± . 84	1.69 ± . 65	0.193
n (%) low PA	20 (20)	4 (13)	16 (23)	–	17 (25)	3 (9)	–
n (%) medium PA	19 (19)	4 (13)	15 (22)	–	15 (22)	4 (13)	–
n (%) high PA	61 (61)	23 (74)	38 (55)	–	36 (53)	25 (78)	–
PAF ng/mL^b^	7.96 (3.89 – 16.77)	9.95 (4.31 – 15.33)	6.45 (3.81 – 18.90)	0.814	4.84 (3.24 – 14.57)	13.27 (9.59 – 21.63)	** < 0.001**
Lp-PLA_2_ nmol/min/mL	14.91 ± 4.29	16.98 ± 4.90	13.98 ± 3.65	** < 0.001**	15.30 ± 4.42	14.09 ± 3.94	0.19
hsCRP mg/L^b,c^	0.96 (0.49 – 2.98)	0.93 (0.41–2.1)	1.1 (0.5–3.14)	0.392	1.79 (0.64 – 3.80)	0.56 (0.22 – 1.01)	** < 0.001**
DASH Index (0–80)	43.86 ± 8.59	43.00 ± 8.44	44.25 ± 8.69	0.505	42.10 ± 8.68	47.59 ± 7.19	**0.002**
Vegetarian Lifestyle Index (0–15)	7.64 ± 1.58	7.08 ± 1.29	7.89 ± 1.65	**0.01**	7.40 ± 1.53	8.15 ± 1.58	**0.026**
HEIFA Score (0–100)	60.04 ± 11.61	60.84 ± 11.73	59.68 ± 11.63	0.647	57.74 ± 11.28	64.94 ± 10.92	**0.003**
MEDAS Score (0–14)	6.45 ± 2.30	6.45 ± 2.43	6.45 ± 2.26	0.996	6.07 ± 2.09	7.25 ± 2.55	**0.016**
erMedDiet Score (0–17)	8.30 ± 2.25	8.10 ± 2.12	8.39 ± 2.31	0.547	8.03 ± 2.02	8.88 ± 2.61	0.112

^a^Independent T-test performed P < 0.05 represents significant difference ^b^ Mann Whitney U test performed P < 0.05 represents significant difference, ^c^ n = 99

BMI, body mass index; DASH, Dietary Approach to Stop Hypertension; erMedDIET, Predimed-Plus Diet Score; HEIFA, Healthy Eating Index for Australian Adults; hsCRP, high-sensitivity c reactive protein; Lp-PLA2, lipoprotein-associated phospholipase A2; mg/L, MEDAS, Mediterranean Diet Adherence Screener; milligrams per litre; ng/L, nanograms per litre; nmol/min/mL, nanomoles per min per millilitre; PA, physical activity; PAF, platelet activating factor; SBP, systolic blood pressure; SD, standard deviation

Bold values indicate statistical significance at *p* < 0.05

### Inflammatory marker results

#### PAF

Median PAF level was 7.96 (3.89–16.77) ng/mL (Table 1). No significant difference was seen between males and females. Median PAF was higher for those at low-risk of CVD compared to those at high-risk (13.27 [9.59–21.63] ng/mL vs. 4.84 [3.24–14.57] ng/mL, *p* < 0.001).

#### Lp-PLA_2_

Mean circulating levels of Lp-PLA_2_ activity were 14.91 ± 4.29 nmol/min/mL and were significantly higher in males than females (16.98 ± 4.90 nmol/min/mL vs. 13.98 ± 3.65 nmol/min/mL, *p* < 0.001). There was no significant difference in Lp-PLA_2_ activity between those at low risk versus high risk of CVD.

#### hsCRP

Median hsCRP levels were 0.96 (0.49–2.98) mg/L (Table 1). No significant difference was seen between males and females however hsCRP levels were higher in those at high risk of CVD 1.79 (0.64–3.80) mg/L compared to those at low risk 0.56 (0.22–1.01) mg/L, *p* < 0.001.

#### Diet Scores

All diet scores significantly correlated with each other with correlations ranging from medium to strong as shown in Table 2. The mean biomarker concentration according to each quartile of diet score is shown in Additional file 1: Table S7.

**Table 2:** Pearson Correlation between Diet Scores

Scores*	DASH Index, r	Dairy-adjusted DASH Index, r	Vegetarian Lifestyle Index, r	HEIFA, r	MEDAS, r
DASH Index					
Vegetarian Lifestyle Index	0.410	0.421			
HEIFA	0.600	0.624	0.381		
MEDAS	0.526	0.541	0.333	0.455	
erMedDiet score	0.489	0.515	0.239	0.419	0.800

*All *p* values < 0.001. r values 0.1–0.29, small correlation; 0.30–0.50, medium correlation; 0.51 – 1, large correlation [59]

DASH, Dietary Approach to Stop Hypertension; erMedDIET, Predimed-Plus Diet Score; HEIFA, Healthy Eating Index for Australian Adults; MEDAS, Mediterranean Diet Adherence Screener

As shown in Table 1, mean diet scores were DASH 43.86 ± 8.59, Vegetarian Lifestyle Index 7.64 ± 1.58, HEIFA 60.04 ± 11.61, MEDAS 6.45 ± 2.30 and erMedDiet Score 8.30 ± 2.25. There was no significant difference in scores between males and females for any of the scores except for the Vegetarian Lifestyle Index where females reported higher mean scores 7.89 ± 1.65 vs. 7.08 ± 1.29. The high-risk group reported significantly lower mean values for all diet scores compared to the lower risk group except for erMedDiet Score.

#### PAF and Diet Scores

No significant correlations were seen for any of the diet scores and logPAF (Tables 3 and 4). Results stratified based on year of data collection are shown in Table 5.

**Table 3:** Correlations between dietary pattern adherence and markers of inflammation

Diet Score	Log PAF		Lp-PLA_2_		Log hsCRP	
	r	*P value*	r	*P value*	r	*P value*
DASH Index	0.124	0.217	− 0.150	0.136	− 0.365	** < 0.001**
Dairy-adjusted DASH Index	0.163	0.104	− 0.141	0.162	− 0.386	** < 0.001**
Vegetarian Lifestyle Index	0.037	0.716	− 0.226	**0.024**	− 0.341	**0.001**
HEIFA	0.110	0.277	− 0.009	0.929	− 0.247	**0.014**
MEDAS	0.127	0.207	− 0.064	0.527	− 0.276	**0.006**
erMedDiet score	0.141	0.162	− 0.104	0.303	− 0.192	0.057

DASH, Dietary Approach to Stop Hypertension; erMedDIET, Predimed-Plus Diet Score; HEIFA, Healthy Eating Index for Australian Adults; Lp-PLA2, lipoprotein-associated phospholipase A2; MEDAS, Mediterranean Diet Adherence Screener; PAF, platelet activating factor

Bold values indicate statistical significance at *p* < .05

**Table 4:** Multiple Linear Regression analysis of the associations between dietary scores and markers of inflammation

	DASH Index		Dairy-adjusted DASH Index		Vegetarian Lifestyle Index^a^		HEIFA		MEDAS		erMedDiet score	
	β	P	β	P	β	P	β	P	β	P	β	P
**LogPAF**												
Model 1	0.047	0.413	0.052	0.374	0.069	0.238	0.086	0.138	0.108	0.280	0.037	0.524
Model 2	0.029	0.624	0.032	0.596	0.007	0.919	0.066	0.273	− 0.002	0.967	0.041	0.469
**Lp-PLA**_**2**_												
Model 1	− 0.126	0.194	− 0.127	0.194	− 0.161	0.105	− 0.008	0.927	− 0.064	0.513	− 0.091	0.349
Model 2	− 0.068	0.515	− 0.066	0.528	− 0.085	0.481	0.064	0.542	− 0.005	0.963	− 0.061	0.536
**LogCRP**												
Model 1	− 0.352	** < 0.001**	− 0.362	** < 0.001**	− 0.394	** < 0.001**	− 0.216	**0.030**	− 0.245	**0.013**	− 0.178	**0.072**
Exp of β^b^	0.703	**–**	0.696	**–**	0.674	–	0.806	-	0.783	–	0.837	–
Model 2	− 0.166	**0.036**	− 0.168	**0.035**	− 0.033	0.725	− 0.010	0.904	− 0.052	0.512	− 0.097	0.203

Model 1: adjusted for age, gender and year of data collection. Model 2: adjusted for age, gender, year of data collection, physical activity, waist circumference, and risk of CVD

^a^Model 2 was adjusted for age, gender, year of data collection, waist circumference and risk of CVD as physical activity was assessed within the score

^b^ Exponentiation of β coefficient; Abbreviations: DASH, Dietary Approach to Stop Hypertension; erMedDIET, Predimed-Plus Diet Score; HEIFA, Healthy Eating Index for Australian Adults; Lp-PLA2, lipoprotein-associated phospholipase A2; MEDAS, Mediterranean Diet Adherence Screener; PAF, platelet activating factor

Bold values indicate statistical significance at *p* < .05

**Table 5:** Multiple Linear Regression analysis stratified by year of data collection of the associations between dietary scores and PAF and Lp-PLA_2_

Novel inflammatory marker/ year of data collection	DASH Index		Dairy-adjusted DASH Index		Vegetarian Lifestyle Index^a^		HEIFA		MEDAS		erMedDiet score	
	β	P	β	P	β	P	β	P	β	P	β	P
**LogPAF2021**												
Model 1	0.182	0.241	0.187	0.233	0.091	0.554	0.189	0.255	0.014	0.927	0.153	0.335
Model 2	0.180	0.255	0.181	0.258	− 0.092	0.592	0.170	0.303	− 0.060	0.732	0.160	0.320
**LogPAF2022**												
Model 1	− 0.076	0.586	− 0.067	0.629	0.146	0.318	0.147	0.320	0.024	0.860	− 0.014	0.918
Model 2	− 0.125	0.422	− 0.114	0.462	0.148	0.458	0.097	0.552	0.024	0.866	− 0.001	0.997
**Lp-PLA**_**2**_**2021**												
Model 1	− 0.200	0.187	− 0.198	0.196	− 0.135	0.369	0.043	0.794	− 0.016	0.917	− 0.062	0.691
Model 2	− 0.157	0.332	− 0.152	0.355	− 0.050	0.778	0.090	0.598	0.038	0.832	− 0.057	0.729
**Lp-PLA**_**2**_**2022**												
Model 1	− 0.042	0.756	− 0.041	0.762	− 0.167	0.243	0.021	0.887	− 0.065	0.622	− 0.082	0.538
Model 2	0.029	0.848	0.029	0.847	− 0.109	0.550	0.066	0.680	− 0.021	0.878	− 0.064	0.641

Model 1: adjusted for age and gender; Model 2: adjusted for age, gender, physical activity, waist circumference, and risk of CVD. ^a^Model 2 was adjusted for age, gender, waist circumference and risk of CVD as physical activity was assessed within the score

#### Lp-PLA_2_ and Diet Scores

There was a small negative correlation between the Vegetarian Lifestyle Index and Lp-PLA_2_ however no significant association remained in either adjusted model. There were no associations found for the other five diet scores. Results stratified based on year of data collection are shown in Table 5.

#### hsCRP and Diet Scores

There was a significant medium negative correlation between logCRP and DASH Index, Dairy-adjusted DASH Index, and Vegetarian Lifestyle Index and a small negative correlation with HEIFA and MEDAS. In model 1 adjusting for age, gender, and year of data collection, the significant medium correlations between logCRP and DASH Index, Dairy-adjusted DASH Index and Vegetarian Lifestyle Index remained and there was a significant small negative correlation with HEIFA, MEDAS and erMedDiet Score. In the second model, which adjusted for age, gender, year of data collection, waist circumference, physical activity and level of risk, only a small negative correlation remained with DASH Index and Dairy-adjusted DASH Index.

As shown in Table 4, a one-point increase in adherence to the DASH Index, the Dairy-adjusted DASH Index and the Vegetarian Lifestyle Index was associated with a 30%, 30%, and 33% reduction in hsCRP levels, respectively. Smaller effects were seen with the other diet scores with a one-point increase in adherence resulting in a 19%, 22% and 16% reduction in hsCRP with HEIFA, MEDAS, erMedDiet scores, respectively.

## Discussion

This cross-sectional study examined the relationship between six diet quality scores (DASH Index, Dairy-adjusted DASH Index, Vegetarian Lifestyle Index, HEIFA, MEDAS and erMedDiet Score) and novel markers of inflammation PAF and Lp-PLA_2_ and hsCRP in 100 Australian adults at varying levels of risk of CVD. It is the first study to examine various healthy dietary patterns using strict exclusion criteria and analysing PAF and Lp-PLA_2_ activity in a broadly Caucasian population. Although the novel biomarkers lacked the expected associations with dietary quality scores, the key finding from this study was that around a 1/3 reduction in hsCRP was predicted by an incremental increase in adherence to the DASH, Dairy-adjusted DASH and Vegetarian Lifestyle Index. This is encouraging as it suggests small improvements in diet may make a large contribution to address inflammatory CVDs. These findings support previous research which found adherence to healthy diets is associated with lower levels of inflammation [60].

Adherence to the Vegetarian Lifestyle Index was shown to be associated with lower levels of Lp-PLA_2,_ and hsCRP in unadjusted and hsCRP in adjusted models, which was unsurprising due to the numerous bioactive compounds found in plants shown to modulate inflammation [61]. However, the lack of association between Lp-PLA_2_ and PAF is surprising and is in contrast to findings from a recent systematic review [26]. These unexpected results could be due to confounding of the COVID-19 vaccine and Omicron outbreak in Australia during data collection. Briefly, PAF and Lp-PLA_2_ are thought to be affected by COVID-19 and its related vaccines as shown in previous research including some mechanistic studies [9, 62–66]. Levels of PAF in the current study significantly differed based on the year of blood sample collection, with higher levels seen in 2022, which coincided with the Omicron variant COVID-19 outbreak in Australia, and a boost in vaccination rates with adenovirus vector and mRNA vaccines. Higher levels of PAF were seen in the low-risk group, the majority of whom were recruited in 2022, whereas 65% of the higher risk group were recruited in 2021 when vaccination and diagnosed COVID-19 rates were low. Although vaccination status was not collected, the vaccination rate in the Gold Coast region was 90.9% for first dose and 88.5% for second dose by the end of January 2022 (unchanged by April 2022), which was the time frame for 2022 data collection [67]. Similarly, there was no difference between the high-risk and low-risk groups for Lp-PLA_2_, suggesting Lp-PLA_2_ levels could also have been elevated due to COVID-19 vaccine and/or infection. This phenomenon is described in more detail elsewhere [9, 62–66]. Attempts were made to control for this effect through our regression models however, adjustment for confounding by a particular variable does not always remove all confounding by that variable and there may be residual confounding [68]. Nevertheless, plant-based dietary interventions have been shown to lower levels of Lp-PLA_2_ in a high risk population [69], and a recent review has highlighted that numerous foods included in a vegetarian diet such as vegetables, eggs, cheese, and animal- and plant-based yoghurts plus plant nutrients such as carotenoids, vitamins C and E, have antithrombotic activity against PAF [70].

The relationship between Vegetarian Lifestyle Index and CRP was strengthened after adjusting for age, gender and year of data collection. However, this relationship disappeared when controlling for other variables such as risk of CVD and waist circumference. This could be due to CVD risk and waist circumference acting as mediators rather than confounders as poor diet may lead to higher waist circumference and increased CVD risk, ultimately leading to inflammation [71–73]. The results of the current study are supported by two recent systematic reviews finding significant negative associations between vegetarian diets and inflammatory markers including CRP [24, 74]. The significant results of the current study could be due to the overall increased number and variety of plant foods consumed, each of which have different bioactive and coloured pigments, many of which have been shown to lower inflammation and confer health benefits [75].

Adherence to the DASH diet was shown to be associated with lower levels of hsCRP in unadjusted and adjusted models but not with PAF or Lp-PLA_2_. The lack of association with PAF and Lp-PLA_2_ and DASH Index was unexpected [26] however this could be due to the COVID-19 confounding previously mentioned and reported elsewhere [9, 76, 77]. The results for hsCRP in the current study align with a systematic review of 7 DASH trials [23].

Interestingly, our sub analysis of DASH where we removed the requirement for 75% of dairy consumed to be low fat, showed a strengthened relationship with hsCRP. This aligns with other research, including a recent prospective cohort study whereby saturated fats from dairy were associated with lower cardiometabolic risk and CRP, [78] and a randomised crossover trial of DASH vs dairy-modified DASH, where the dairy-modified DASH was as effective as DASH in reducing blood pressure and lipids [79]. Our findings also support that the fat in dairy may be less inflammatory than previously thought [80–85]. This is an area for ongoing research, given the continued message to consume low fat dairy products in global dietary guidelines [86–88], which may require revision.

Surprisingly, given previous research on PAF and the Mediterranean diet, [26, 89, 90] there was no association between MEDAS and erMedDietScore and PAF and Lp-PLA_2_. The small association between hsCRP and HEIFA, MEDAS and erMedDiet Score was also unexpected due to previous research showing diets consistent with country-specific guidelines and Mediterranean diets were associated with significantly lower levels of this marker [25, 91]. This could be due to the AGHE requiring fewer fruit serves and not prioritising legumes and nuts, both of which are emphasised in the DASH Index and Vegetarian Lifestyle Index, and which showed stronger relationships in the current study. In addition, a previous MEDAS study found that the food groups emphasised in the DASH dietary pattern had the strongest association with CRP [92].

### Strengths and limitations

Strengths of this study include the strict exclusion criteria to prevent confounding and the use of validated FFQ and diet quality scores for assessing diet.

However, there were some limitations. Dietary assessment is difficult and prone to error. FFQs overestimate some food groups like fruit and vegetables [93]. Some of the scores did not adjust for energy intake potentially confounding results as higher consumption of energy can result in higher intakes generally and therefore scores. PAF and Lp-PLA_2_ levels may have been elevated in some of the participants due to the COVID-19 vaccine and/or infection [9]. Further, PAF and Lp-PLA_2_ may be associated with more severe forms of CVD and this study’s sample only included disease-free individuals.

## Conclusion

In conclusion, this study found that higher adherence to several dietary patterns was associated with lower levels of inflammation as measured by hsCRP, with the strongest relationship seen with Vegetarian Lifestyle Index, DASH Index and Dairy-adjusted DASH Index. The novel inflammatory marker findings were unexpected with no association in adjusted models for either Lp-PLA_2_ or PAF, possibly confounded by COVID-19 infections and/or vaccinations. Future research should aim to examine the relationship with these novel markers and healthy dietary patterns in larger, more diverse samples and in a non-pandemic setting.

### Supplementary Information

**Additional file 1**. Dietary intake scoring details, flow of study participants, and table of unadjusted values for biomarker concentrations according to quartile of diet score.

## Data Availability

Data described in the manuscript, code book, and analytic code will be made available upon request pending application and approval. Requests should be emailed to the corresponding author.

## Abbreviations

CVD: Cardiovascular disease
DASH: Dietary approach to stop hypertension
erMED: Predimed-Plus Diet Score
FETA: FFQ EPIC tool for analysis
HEIFA: Healthy Eating Index for Australian
hsCRP: High-sensitivity C-reactive protein
Il-6: Interleukin 6
Lp-PLA_2_: Lipoprotein-associated phospholipase A2
LysoPC: Lysophophatidylcholine
MEDAS: Mediterranean diet adherence screener
oxNEFA: Oxidized, non-esterified fatty acids
PAF: Platelet-activating factor
TNF-α: Tumour necrosis factor alpha
