# Correction: The association between dietary quality scores with C-reactive protein and novel biomarkers of inflammation platelet-activating factor and lipoprotein-associated phospholipase A2: a cross-sectional study

**DOI:** 10.1186/s12986-023-00771-y

**Published:** 2023-11-27

**Authors:** Carolyn J. English, Anna E. Lohning, Hannah L. Mayr, Mark Jones, Helen MacLaughlin, Dianne P. Reidlinger

**Affiliations:** 1 Faculty of Health Sciences and Medicine, Bond University, Robina, QLD Australia; 2 Department of Nutrition and Dietetics, Princess Alexandra Hospital, Woolloongabba, QLD Australia; 3 Centre for Functioning and Health Research Metro South Hospital and Health Service, Brisbane, QLD Australia; 4 Faculty of Health Sciences and Medicine, Institute of Evidence-Based Healthcare, Bond University, Robina, QLD Australia; 5 Faculty of Health, School of Exercise and Nutrition Sciences, Queensland University of Technology, Brisbane, Australia; 6 Nutrition Research Collaborative, Royal Brisbane and Women’s Hospital, Brisbane, Australia

**Correction: Nutrition & Metabolism (2023) 20:38** **https://doi.org/10.1186/s12986-023-00756-x**

Following the publication of the original article [1], the authors identified errors in the description of the score range of the Vegetarian Lifestyle Index in Table 1 and in the Methods section. The updated sentence and Table 1 are given below, and the changes have been highlighted in **bold** **typeface**.

The sentence currently reads:

The total vegetarian score was calculated by summing all of the points from each respective section to generate a composite score ranging in values from 0 to 80.

The sentence should read:

The total vegetarian score was calculated by summing all of the points from each respective section to generate a composite score ranging in values from 0 to **14**.

The incorrect Table 1 is:

**Table 1:** Demographic and clinical characteristics of study subjects

Characteristics	Mean ± SD or N (%) or Median (IQR range)				Mean ± SD or N (%) or Median (IQR range)		
	Total n = 100	Male n = 31	Female n = 69	*P value*^*a*^	High risk of CVD n = 68	Low risk of CVD n = 32	***P value***^***a***^
Age, years^b^	49 ± 13	46 ± 13	50 ± 13	0.120	53 ± 13	38 ± 14	** < 0.001**
Race, Caucasian n (%)	92 (92)	25 (86)	67 (94)	−	65 (96)	27 (84)	–
Male n (%)	31 (31)			−	21 (31)	10 (31)	–
BMI, kg/m^2b^	28.3 ± 6.5	27.41 ± 5.0	28.65 ± 7.2	0.729	30.65 ± 6.4	23.19 ± 2.7	** < 0.001**
Waist Circumference (cm) Umbilicus^b^	95.8 ± 6.7	95.99 ± 12.60	95.70 ± 18.40	0.526	102.36 ± 15.40	81.83 ± 9.15	** < 0.001**
Type 2 Diabetes diagnosis %	4 (4)	3 (10)	1 (1)	–	4 (6)	0 (0)	–
Physical Activity METs tertiles	1.41 ± 0.65	1.61 ± 0.72	1.32 ± 0.83	0.193	1.28 ± . 84	1.69 ± . 65	0.193
n (%) low PA	20 (20)	4 (13)	16 (23)	–	17 (25)	3 (9)	–
n (%) medium PA	19 (19)	4 (13)	15 (22)	–	15 (22)	4 (13)	–
n (%) high PA	61 (61)	23 (74)	38 (55)	–	36 (53)	25 (78)	–
PAF ng/mL^b^	7.96 (3.89 – 16.77)	9.95 (4.31 – 15.33)	6.45 (3.81 – 18.90)	0.814	4.84 (3.24 – 14.57)	13.27 (9.59 – 21.63)	** < 0.001**
Lp-PLA_2_ nmol/min/mL	14.91 ± 4.29	16.98 ± 4.90	13.98 ± 3.65	** < 0.001**	15.30 ± 4.42	14.09 ± 3.94	0.19
hsCRP mg/L^b,c^	0.96 (0.49 – 2.98)	0.93 (0.41–2.1)	1.1 (0.5–3.14)	0.392	1.79 (0.64 – 3.80)	0.56 (0.22 – 1.01)	** < 0.001**
DASH Index (0–80)^d^	43.86 ± 8.59	43.00 ± 8.44	44.25 ± 8.69	0.505	42.10 ± 8.68	47.59 ± 7.19	**0.002**
Vegetarian Lifestyle Index (0–15)^d^	7.64 ± 1.58	7.08 ± 1.29	7.89 ± 1.65	**0.01**	7.40 ± 1.53	8.15 ± 1.58	**0.026**
HEIFA Score (0–100)^d^	60.04 ± 11.61	60.84 ± 11.73	59.68 ± 11.63	0.647	57.74 ± 11.28	64.94 ± 10.92	**0.003**
MEDAS Score (0–14)^d^	6.45 ± 2.30	6.45 ± 2.43	6.45 ± 2.26	0.996	6.07 ± 2.09	7.25 ± 2.55	**0.016**
erMedDiet Score (0–17)^d^	8.30 ± 2.25	8.10 ± 2.12	8.39 ± 2.31	0.547	8.03 ± 2.02	8.88 ± 2.61	0.112

^a^Independent T-test performed P < 0.05 represents significant difference ^b^ Mann Whitney U test performed P < 0.05 represents significant difference, ^c^ n = 99, ^d^ Higher scores indicate greater adherence

BMI, body mass index; DASH, Dietary Approach to Stop Hypertension; erMedDIET, Predimed-Plus Diet Score; HEIFA, Healthy Eating Index for Australian Adults; hsCRP, high-sensitivity c reactive protein; Lp-PLA2, lipoprotein-associated phospholipase A2; mg/L, MEDAS, Mediterranean Diet Adherence Screener; milligrams per litre; ng/L, nanograms per litre; nmol/min/mL, nanomoles per min per millilitre; PA, physical activity; PAF, platelet activating factor; SBP, systolic blood pressure; SD, standard deviation

Bold values indicate statistical significance at *p* < .05

The correct Table 1 is:

**Table 1:** Demographic and clinical characteristics of study subjects

Characteristics	Mean ± SD or N (%) or Median (IQR range)			*P* value^a^	Mean ± SD or N (%) or Median (IQR range)		*P* value^a^
	Total n = 100	Male n = 31	Female n = 69		High Risk of CVD n = 68	Low Risk of CVD n = 32	
Age, years^b^	49 ± 13	46 ± 13	50 ± 13	120	53 ± 13	38 ± 14	** < 0.001**
Race, Caucasian n (%)	92 (92)	25 (86)	67 (94)		65 (96)	27 (84)	
Male n (%)					21 (31)	10 (31)	
BMI, kg/m^2b^	28.3 ± 6.5	27.41 ± 5.0	28.65 ± 7.2	0.729	30.65 ± 6.4	23.19 ± 2.7	** < 0.001**
Waist Circumference (cm) Umbilicus^b^	95.8 ± 6.7	95.99 ± 12.60	95.70 ± 18.40	0.526	102.36 ± 15.40	81.83 ± 9.15	** < 0.001**
Type 2 Diabetes diagnosis %	4 (4)	3 (10)	1 (1)	–	4 (6)	0 (0)	–
Physical Activity METs tertiles	1.41 ± 0.65	1.61 ± 0.72	1.32 ± 0.83	0.193	1.28 ± . 84	1.69 ± . 65	0.193
n (%) low PA	20 (20)	4 (13)	16 (23)	–	17 (25)	3 (9)	–
n (%) medium PA	19 (19)	4 (13)	15 (22)	–	15 (22)	4 (13)	–
n (%) high PA	61 (61)	23 (74)	38 (55)	–	36 (53)	25 (78)	–
PAF ng/mL^b^	7.96 (3.89–16.77)	9.95 (4.31–15.33)	6.45 (3.81–18.90)	0.814	4.84 (3.24–14.57)	13.27 (9.59–21.63)	** < 0.001**
Lp-PLA_2_ nmol/min/mL	14.91 ± 4.29	16.98 ± 4.90	13.98 ± 3.65	** < 0.001**	15.30 ± 4.42	14.09 ± 3.94	0.19
hsCRP mg/L^b,c^	0.96 (0.49–2.98)	0.93 (0.41–2.1)	1.1 (0.5–3.14)	0.392	1.79 (0.64–3.80)	0.56 (0.22–1.01)	** < 0.001**
DASH Index (0–80)	43.86 ± 8.59	43.00 ± 8.44	44.25 ± 8.69	0.505	42.10 ± 8.68	47.59 ± 7.19	**0.002**
Vegetarian Lifestyle Index (0–14)	7.64 ± 1.58	7.08 ± 1.29	7.89 ± 1.65	**0.01**	7.40 ± 1.53	8.15 ± 1.58	**0.026**
HEIFA Score (0–100)	60.04 ± 11.61	60.84 ± 11.73	59.68 ± 11.63	0.647	57.74 ± 11.28	64.94 ± 10.92	**0.003**
MEDAS Score (0–14)	6.45 ± 2.30	6.45 ± 2.43	6.45 ± 2.26	0.996	6.07 ± 2.09	7.25 ± 2.55	**0.016**
erMedDiet Score (0–17)	8.30 ± 2.25	8.10 ± 2.12	8.39 ± 2.31	0.547	8.03 ± 2.02	8.88 ± 2.61	0.112

*BMI* body mass index, *DASH* Dietary Approach to Stop Hypertension, *erMedDIET* Predimed-Plus Diet Score, *HEIFA* Healthy Eating Index for Australian Adults, *hsCRP* high-sensitivity c reactive protein, *Lp-PLA2* lipoprotein-associated phospholipase A2, *mg/L*, *MEDAS*, Mediterranean Diet Adherence Screener; milligrams per litre, *ng/L*, nanograms per litre, *nmol/min/mL* nanomoles per min per millilitre, *PA* physical activity, *PAF* platelet activating factor, *SBP* systolic blood pressure, *SD* standard deviation

^a^Independent T-test performed **P < *0.05*** represents significant difference

^b^Mann Whitney U test performed ***P* < *0.05*** represents significant difference

^c^n = 99

The original article [1] has been corrected.
