# Supplementary material for: The association between dietary quality scores with C-reactive protein and novel biomarkers of inflammation platelet-activating factor and lipoprotein-associated phospholipase A2: a cross-sectional study
Source: Nutr Metab (Lond). 2023 Sep 12;20:38. doi: 10.1186/s12986-023-00756-x (PMC10496320; doi:10.1186/s12986-023-00756-x)
Supplement: Supplementary file 1 — Additional file 1. Dietary intake scoring details, flow of study participants, and table of unadjusted values for biomarker concentrations according to quartile of diet score. [file 12986_2023_756_MOESM1_ESM.docx]

**The association between dietary quality scores with C-reactive protein and novel biomarkers of inflammation platelet-activating factor and lipoprotein-associated phospholipase A2**

**Supplementary Files**

English CE, Lohning AE, Mayr HL, Jones M, MacLaughlin H, Reidlinger DP, The association between dietary quality scores with C-reactive protein and novel biomarkers of inflammation platelet-activating factor and lipoprotein-associated phospholipase A2: A cross-sectional study. Submitted to Nutrition & Metabolism, 2023.

Supplementary Table 1 DASH Diet Index Scoring^(56)^

| Dietary Component | Maximum Score | Standard for Maximum Score | Standard for Minimum Score |
| --- | --- | --- | --- |
| Fruit | 10 | ≥4 servings/day | 0 servings/day |
| Vegetables | 10 | ≥4 servings/day | 0 servings/day |
| Grains |  |  |  |
| Total | 5 | ≥6 servings/day | 0 servings/day |
| High Fiber | 5 | ≥50% of daily servings | 0% servings/day |
| Dairy |  |  |  |
| Total | 5 | ≥2 servings/day | 0 servings/day |
| Low Fat | 5 | ≥75% of daily servings | 0% servings/day |
| Meat, poultry, fish, eggs | 10 | ≤ 2 servings/day | ≥4 servings/day |
| Nuts, seeds, legumes | 10 | ≥4 servings/week | 0 servings/week |
| Fats, oils | 10 | ≤3 servings per day | ≥6 servings/day |
| Sweets | 10 | ≤5 servings/week | ≤5 servings/week |

Supplementary Table 2 Vegetarian Lifestyle Index Scoring^(60)^

| **Diet component** | **Description** | **Recommendation** | **Operationalisation** | **Score** |
| --- | --- | --- | --- | --- |
| Whole grains | Whole grain bread, cereals such as oatmeal, and brown rice | 6 servings/day | <3 servin*g*s/day | 0 |
|  |  |  | ≥3 and <6 servings/day | 0.5 |
|  |  |  | ≥6 servings/day | 1 |
| Legumes, soy and meat substitutes | Beans, peas, soy, and meat substitutes | 3 servings /day | <1 servings/day | 0 |
|  |  |  | ≥1 and <3 servings/day | 0.5 |
|  |  |  | ≥3 serving/day | 1 |
| Vegetables | Dark green, avocado, and 100% vegetables juices | 8 servings/day | < 4 servings/day  ≥ 4 and <8 servings/day  ≥ 8 servings /day | 0  0.5  1 |
| Fruit | Fresh and dried fruits, canned or cooked fruits, and 100% fruit juices | 4 servings/day | < 2 servings/day  ≥ 2 and <4 servings/day  ≥ 4 servings/day | 0  0.5  1 |
| Nuts and seeds | Nuts and seeds (raw or roasted) | 1-2 servings/day | < 4 servings/week  ≥ 4 servings/week and <1.5 servings /day  ≥1.5 servings/day | 0  0.5  1 |
| Vegetable oils | Olive oil, and other salad oils | 2 servings/day | > 4 servings/day  >2 and ≤ 4 servings/day  0-2 servings/day | 0  0.5  1 |
| Dairy | Dairy products, cheese, milk, and yoghurt | 2 servings/day | <0.5 or >2 servings/day  ≥ 0.5 and < 1 servings/day  1-2 servings/day | 0  0.5  1 |
| Eggs | Fried, boiled, scrambled, devilled, plain omelette, egg salad | ½ servings/day | >1 serving/day  >0.5 and ≤ 1 servings/day  ≥0 and ≤0.5 servings/day | 0  0.5  1 |
| Sweets | Sweets, sugary beverages, and desserts | Consume sweets and sugary beverages sparingly | >5 servings/week  >2 and ≤ 5 servings/week  0-2 servings/week | 0  0.5  1 |
| Reliable source vitamin B12 | Reliable sources of vitamin B-12 from reliable sources including meat, fish, dairy, eggs, yeast, fortification (cereals, meat substitutes, soymilk) supplements | Meeting the recommended (EAR) of 2.0 mcg daily of vitamin B-12 | <1.0 mcg serving equivalent/day  ≥1.0 and <2.0 mcg serving equivalent/day  ≥ 2.0 mcg serving equivalent/day | 0  0.5  1 |
| Flesh-food intake | Red meat, processed meat, poultry, and fish |  | >1 time/week  ≤1 time/week and > 1 time/month  ≤1 time a month | 0  0.5  1 |
| **Lifestyle component** | | | | |
| Daily exercise | Moderate/rigorous physical activity | 30 min/day to avoid chronic disease, and 60 min/day for weight loss | ≤0 min/day of moderate OR ≤0 min/day vigorous exercise  >0 and <30min/day of moderate exercise OR >0 and <15 min/day of vigorous exercise  ≥30 min/day of moderate  OR ≥15min/day of vigorous exercise | 0  0.5  1 |
| Water intake | Drinking water | At least eight, 250 ml glasses of water daily | <4 glasses of water/day  ≥4 and <8 glasses of water/day  ≥8 glasses of water/day | 0  0.5  1 |
| Sunlight exposure | Adequate exposure to sunlight | At least 10 min of sun a day to activate vitamin D | <5 min/day  >5 and <10 min/day  ≥10 min/day | 0  0.5  1 |

Supplementary Table 3 HEIFA Scoring^(62)^

| Dietary guidelines for Australian adults | Indicator and description | Criteria for maximum score | Criteria for minimum score | Composite subscores |
| --- | --- | --- | --- | --- |
| To achieve and maintain a healthy weight, be physically active, and choose amounts of nutritious foods and drinks to meet energy needs | Measure of unhealthy choices, discretionary foods : Frequency of consumption of extra foods per day e.g., 1 slice (40 g) plain cake or small cake-type muffin | Discretionary foods:  M ≤3, F≤ 2.5 | Discretionary foods:  M≥3, F≥2.5 | Discretionary foods/d:  Male:  <3 serves/d =10  3–3.9 =7.5  4.0–4.9 =5  5–5.9 =2.5  6 serves =0  Female:  <2.5 serves/d = 10  2.5–3.4 = 7.5  3.5–4.4 = 5  4.5–5.4 = 2.5  5.5 = 0 |
| Eat plenty of vegetables of different types and colours, and legumes/beans | Total vegetables: servings of vegetables and legumes per day e.g., 1 (75 g) medium tomato Variety of vegetable servings/d | M:≥6 serves  F:≥5 serves  Varied vegetables e.g., green, orange, cruciferous, tuber, or bulb and legumes consumed | Total vegetables 0 Variety 0 | Total vegetable serves per day  Male:  ≥6 serves=5  ≥4.8 to<6 serves=4  ≥3.6 to<4.8 serves=3  ≥2.4 to<3.6 serves=2  ≥1.0 to<2.4 serves=1  No vegetables=0  Female:  ≥5 serves=5  ≥4to<5 serves=4  ≥3to<4 serves=3  ≥2to<3 serves=2  ≥1to<2 serves=1  No vegetables=0  Vegetable variety score/d:  ≥1serve green=1  ≥1 serve orange=1  ≥1 serve of cruciferous=1  ≥1 serve of tuber or bulb=1  ≥0.5 serves of legumes=1 |
| Enjoy a wide variety of fruits | Fruit servings per day; variety of fruit servings/d e.g., 1 (150 g) medium apple, banana, orange, or pear | Fruits: 2 or more serves/d  Varied fruits consumed | No fruit, whole fruits, 0  Variety 0 | Total fruit serves/day:  ≥2 serves=5  ≥1.5 to<2 serves=3.75  ≥1to<1.5 serves=2.5  ≥0.5 to<1 serves=1.25  ≥0to<0.5 serves=0  Fruit variety score/d  2 or more varieties 5 points |
| Enjoy a wide variety of grain (cereal) foods, mostly whole-grain and/or high-cereal-fiber varieties such as breads, cereals, rice, pasta, noodles, polenta, couscous, oats, quinoa, and barley | Whole grains Refined grains Cereals: frequency of consumption of breads and cereals per day. Whole-grain cereals: proportion of whole meal/whole-grain bread consumed relative to total bread e.g., 1 slice (40 g) bread | Whole grains:50% whole grains  M≥6, F≥6 | <50% whole grains  M≤6, F≤6 | Total cereals serves/d:6 serves=5  5 serves=4.17  4 serves=3.34  3 serves=2.5  2 serves=1.67  1 serve=0.84  Whole-grain cereal serves/d:  ≥3 serves=5  ≥2.5 to<3 serves=4  ≥2to<2.5 serves=3,  ≥1.5 to<2 serves=2,  ≥1to=1.5 serve=1  No whole grain=0 |
| Enjoy a wide variety of lean meats and poultry, fish, eggs, tofu, nuts, and seeds, and legumes/beans | Total protein foods, seafood and plant proteins, and lean meat and meat alternatives: frequency of consumption of lean meats and alternatives per day e.g., 65 g cooked lean red meat such as beef, lamb. One cup (150 g) cooked or canned legumes/beans | M≥3, F≥2.5 | No protein foods  No seafood or plant proteins 0 | Total cereals serves/d:  6 serves=5  5 serves=4.17  4 serves=3.34  3 serves=2.5  2 serves=1.67  1 serve=0.84  Whole-grain cereal serves/d:  ≥3 serves=5  2.5 to<3 serves=4  ≥2to<2.5 serves=3  ≥1.5 to<2 serves=2  1to≥1.5 serve=1  No whole grain=0 |
| Enjoy a wide variety of milk, yoghurt, cheese, and/or their alternatives mostly reduced fat | Low-fat dairy consumption/ dairy foods/alternatives: frequency of consumption of dairy products per day Low-fat/reduced-fat dairy: type of milk usually consumed e.g., 1 c | M≥2.5, F≥2.5 | No dairy | Dairy/dairy alternatives/d:  ≥2.5 serves=10  ≥2 serves=8  ≥1.5 serves=6  ≥1 serves=4,  ≥0.5 serves=2,  No dairy/dairy alternative=0 |
| Drink plenty of water | Fluids: proportion of water consumed relative to total beverages Includes water/tea/coffee | 50% water consumed relative to total beverages | No water | Proportion of water consumed relative to total beverages/d:  ≥50%=5  ≥40%=4,  ≥30%=3  ≥20%=2  ≥10%=1  0%=0  Note: only scored out of 5 |
| Limit intake of foods high in saturated fat and replace high-fat foods with foods that contain predominately PUFA and MUFA | Consumption of high saturated fat, nutrient low-density foods, and fatty | % energy intake: total saturated fat<10%,  PUFA and MUFA | % energy intake: saturated fat>10%, PUFA and MUFA none Less than 1 serve for men and less than 0.5 serve for women | Saturated fat≤10% of energy=5,>10–12% of energy=2.5>12% total energy=0,PUFA and MUFA  Men  4 serves=5  3 to<4 serves=3.75  2 to<3 serves=2.5  1 to<2 serve=1.25  0 to<1 serve=0  Women  2 serves=5  1.5 to<2serves=3.75  1 to<1.5 serves=2.5  0.5 to<1 serve=1.25  0 to<0.5 serves=0 |
| Choose lower sodium options and do not add salt to foods in cooking or at the table | Sodium options, salt used in cooking and at the table | ≤70 mmol (920 mg) Na/d | ≥100 mmol (2300 mg) of Na/d | Sodium/d:  0 to ≤ 70 mmol (920–1610 mg) = 10, 70 to ≤100 mmol (1610–2300 mg) = 5 ≥100 mmol (2300 mg) of Na/d = 0 |
| Limit intake of foods containing added sugars | Consume only moderate amounts of sugars and foods containing added sugars. Added sugars: frequency of consumption of soft drink, cordial, fruit juice drink, jam, chocolate, confectionary, grain desserts (e.g., cakes, pies), dairy desserts, and candy per day. Percentage of energy from sugar | <1.5 serves<15% of total energy | >1.5 serves>20% of total energy | Serves of foods containing added sugars:<15% total energy=10  >15% to<20% total energy=5  >20% energy=0 |
| If you choose to drink alcohol, limit intake | Alcohol: frequency of consumption of all alcoholic beverages per day 200 mL wine (2 standard drinks) | <2 per day | >2 per day  Only scored out of 5 | ≤2 per day = 5  >2 per day = 0 |

Supplementary Table 4 Mediterranean Diet Adherence Screener Scoring^(63)^

| **Criteria** | **Scoring** |
| --- | --- |
| Use of mainly extra-virgin olive oil for cooking, salad dressings, and spreads | Yes =1  No=0 |
| Quantity (tablespoons) of olive oil consumed in a given day (including oil used for frying, salads, out of house meals, etc.) | ≥4 = 1  <4 =0 |
| Serves of fruit (including dried fruit and natural fruit juices) consumed per day | ≥3 = 1  <3 =0 |
| Serves of vegetables consumed per day (1 serving: ½ cup cooked or 1 cup salad) | ≥6 =1  <6=0 |
| Times per week vegetables, pasta or other dishes seasoned with sofrito (sauce made with tomato and onion, leek or garlic and simmered with olive oil) | ≥2 =1  <2=0 |
| Servings of legumes consumed per week (1 serving: 150g or 1 cup) | ≥3 =1  <3=0 |
| Servings of fish or shellfish consumed per week (1 serving: 100-150g of fish or 4-5 units or 200g shellfish) | ≥3 =1  <3 =0 |
| Servings of nuts consumed per week (1 serving: 30g) | ≥3 =1  <3 =0 |
| Servings of red meat or processed meats (ham, sausage, hamburgers, etc.) consumed per week (1 serving: 100 - 150 g) | ≤1=1  <1 =0 |
| Is chicken or turkey meat instead or red meat, pork or processed meats preferentially consumed | Yes=1  No=0 |
| Servings of sweet/carbonated beverages consumed per week | <1 glass =1  ≥1 =0 |
| Servings of butter, margarine, or cream consumed per week (1 serving: 2 tsp) | <1 =1  ≥1 =0 |
| Times per week commercial sweets or pastries (not homemade), such as cakes, cookies, biscuits or custard consumed | <3 =1  ≥3 =0 |
| Glasses of wine consumed per week (1 glass: 100 ml) | ≥7 =1  <7 =0 |
| TOTAL SCORE OUT OF 14 |  |

Supplementary Table 5 erMedDiet Scoring^(64)^

| **Criteria** | **Scoring** |
| --- | --- |
| Use of mainly extra-virgin olive oil for cooking, salad dressings, and spreads | Yes =1  No=0 |
| Serves of fruit (including dried fruit and natural fruit juices) consumed per day | ≥3 = 1  <3 =0 |
| Serves of vegetables consumed per day (1 serving: ½ cup cooked or 1 cup salad) | ≥6 =1  <6=0 |
| Times per week vegetables, pasta or other dishes seasoned with sofrito (sauce made with tomato and onion, leek or garlic and simmered with olive oil) | ≥2 =1  <2=0 |
| Times per week whole grains, breads or cereals consumed | ≥5 =1  <5=0 |
| Times per week white/refined grains or cereals such as pasta or rice (not including bread) consumed | <3 =1  ≥3=0 |
| Servings of white bread consumed per day (1 serving: 2 slices bread) | ≤1 =1  <1 =0 |
| Servings of legumes consumed per week (1 serving: 150g or 1 cup) | ≥3 =1  <3=0 |
| Servings of fish or shellfish consumed per week (1 serving: 100-150g of fish or 4-5 units or 200g shellfish) | ≥3 =1  <3 =0 |
| Servings of nuts consumed per week (1 serving: 30g) | ≥1 =1  <1 =0 |
| Servings of red meat or processed meats (ham, sausage, hamburgers, etc.) consumed per week (1 serving: 100 - 150 g) | ≤1=1  <1 =0 |
| Is chicken or turkey meat instead or red meat, pork or processed meats preferentially consumed | Yes=1  No=0 |
| Servings of sugary beverages or sugar-sweetened fruit juices consumed per week | <1 glass =1  ≥1 =0 |
| Servings of butter, margarine, or cream consumed per week (1 serving: 2 tsp) | <1 =1  ≥1 =0 |
| Times per week commercial sweets or pastries (not homemade), such as cakes, cookies, biscuits or custard consumed | <3 =1  ≥3 =0 |
| Glasses of wine consumed per day (1 glass: 100 ml) | M:  2-3 =1  <2 or >3=0  W:  1-2 =1  <1 or >2=0 |
| TOTAL SCORE OUT OF 17 | |

Assessed for eligibility (n=132)

Excluded (n=32)

- Not meeting inclusion criteria (n=4)
- Declined to participate (n=28)

Analysed (n=100)

- Excluded from hsCRP analysis only (insufficient blood sample) (n=1)

Attended data collection visit (n=100)

- Data collected in 2021 (n=46)
- Data collected in 2022 (n=54)

## Analysis

## Enrollment

*Supplementary Table 6 Flow of participants through a study investigating the association between dietary quality scores and* *markers of inflammation*

Supplementary Table 7 Unadjusted mean ± SD or median (IQR) biomarker concentration according to quartile of diet score

| **PAF ng/mL** | | | | | **Lp-PLA_2_ nmol/min/mL** | | | | **CRP mg/L** | | | |
| --- | --- | --- | --- | --- | --- | --- | --- | --- | --- | --- | --- | --- |
|  | Q1 | Q2 | Q3 | Q4 | Q1 | Q2 | Q3 | Q4 | Q1 | Q2 | Q3 | Q4 |
| DASH Index | 4.92 (3.64-14.24) | 8.31 (2.70-20.06) | 9.54 (4.34-19.29) | 7.87 (4.34-14.25) | 16.35±4.77 | 13.63±3.54 | 14.82±5.04 | 14.78±3.43 | 2.30 (0.89-4.48) | 1.45 (0.5-3.88) | 0.70 (0.35-2.67) | 0.57 (0.22-1.36) |
| Vegetarian Lifestyle Index | 4.77 (2.47-12.21) | 11.15 (4.88-16.64) | 9.47 (4.34-22.55) | 5.25 (3.56 – 15.08) | 16.99±5.01 | 13.86±2.91 | 14.22±3.84 | 14.57±4.0 | 2.53 (0.94-5.15) | 0.81 (0.48-2.98) | 0.64 (0.29-2.19) | 0.84 (0.39-2.86) |
| HEIFA | 4.87 (2.03-15.0) | 11.26 (6.1-22.33) | 4.85 (3.99-14.41) | 9.05 (4.10-19.98) | 14.89±5.54 | 14.95±4.06 | 14.97±4.30 | 14.83±3.21 | 2.23 (0.83-5.50) | 0.83 (0.28-2.20) | 1.27 (0.55-3.15) | 0.66 (0.44-1.89) |
| MEDAS | 4.88 (3.24-15.26) | 8.05 (2.85-13.66) | 9.54 (4.94-20.01) | 11.18 (4.34-20.09) | 14.81±4.31 | 16.26±5.12 | 15.44±4.72 | 13.62±2.80 | 2.07 (0.81-4.30) | 0.66 (0.50-4.30) | 0.89 (0.54-2.38) | 0.56 (0.21-1.69) |
| erMedDiet score | 7.46 (2.10-16.12) | 4.87 (3.78-15.82) | 5.42 (3.88-15.21) | 13.23 (4.75-19.29) | 15.13±4.49 | 15.55±3.78 | 15.66±4.94 | 13.34±3.17 | 1.79 (0.73-4.65) | 0.93 (0.34-2.62) | 0.67 (0.46-2.32) | 0.74 (0.23-2.05) |

Q4 represents highest adherence to the dietary score. Abbreviations: IQR, interquartile range; mg/L, milligrams per litre ng/L, nanograms per litre; nmol/min/mL, nanomoles per min per millilitre; SD, standard deviation
